# Frontiers in metabolic physiology grand challenges

**DOI:** 10.3389/fphys.2022.879617

**Published:** 2022-08-10

**Authors:** John D. Imig

**Affiliations:** Drug Discovery Center, Medical College of Wisconsin, Milwaukee, WI, United States

**Keywords:** metabolism, glucose, lipids, obesity, diabetes, hepatic disease, nutrition

## Introduction

Metabolic physiology is defined as the molecular, cellular, organ, and organism biological processes that provide energy to maintain homeostasis. Research on metabolism touches on all areas of physiology and examines issues relating to major health problems. Metabolic diseases such as obesity, type 2 diabetes, metabolic syndrome, and liver diseases are increasing at alarming rates (O'Neill and O'Driscoll, 2015; Saklayen, 2018). These metabolic diseases challenge physiologists to utilize technical advances to profile metabolism at all levels. *Frontiers in Metabolic Physiology* was launched in March 2021 and quickly started attracting excellent scientific and review articles that address the major challenges in metabolic physiology.

## Grand challenge: Understanding metabolism and metabolites

Metabolism and metabolites are required for the generating energy at the cellular levels to maintain homeostasis. Critical metabolites include carbohydrates, lipids, and amino acids which generate the cellular chemical carrier of energy, ATP. Organs like the liver precisely regulate glucose and lipid metabolism in healthy individuals (Jones, 2016; Pereira et al., 2021). At the level of the organism energy homeostasis is attained via balancing energy intake and energy expenditure. On the other hand, dysregulation of metabolism at the cellular, organ, and organism levels results in several metabolic diseases including obesity, diabetes, fatty liver disease, and atherosclerotic cardiovascular diseases (Newgard 2017; Saklayen, 2018). A major challenge is identifying novel metabolism and metabolite regulators or pathways at the cellular, organ, and organism levels that maintain energy homeostasis or results in metabolic dysfunction and disease.

There are major research challenges in evaluating the regulation of glucose and lipid metabolism. Glucose is the ubiquitous source of energy for every organism and is essential to fuel both aerobic and anaerobic cellular respiration. Glucose is broken down in a series of biochemical reactions releasing energy in the form of ATP. Likewise, lipids such as cholesterol, phospholipids, triglycerides, and free fatty acids, have several metabolic physiological roles (Ponziani et al., 2015). Metabolic physiology focuses on the liver, pancreas, gastrointestinal track, skeletal muscle, and adipose tissue as organs essential for glucose and lipid metabolism and energy homeostasis. Metabolic processes in the liver include lipogenesis, fatty acid oxidation, gluconeogenesis, cholesterol biosynthesis and elimination, and lipoprotein transport (Ponziani et al., 2015). The pancreas and skeletal muscle are key regulatory sites for insulin and glucose regulation and utilization (Mizgier et al., 2014). Adipose tissue is a highly dynamic, metabolically active tissue that is involved in lipid handling and metabolic homeostasis (Frayn, 2002). Current research challenges include identifying cell signaling pathways contributing to glucose and insulin regulation, determining novel pathways regulating lipid metabolism, and assessing crosstalk between metabolic organs that contribute to energy metabolism.

**FIGURE 1:**
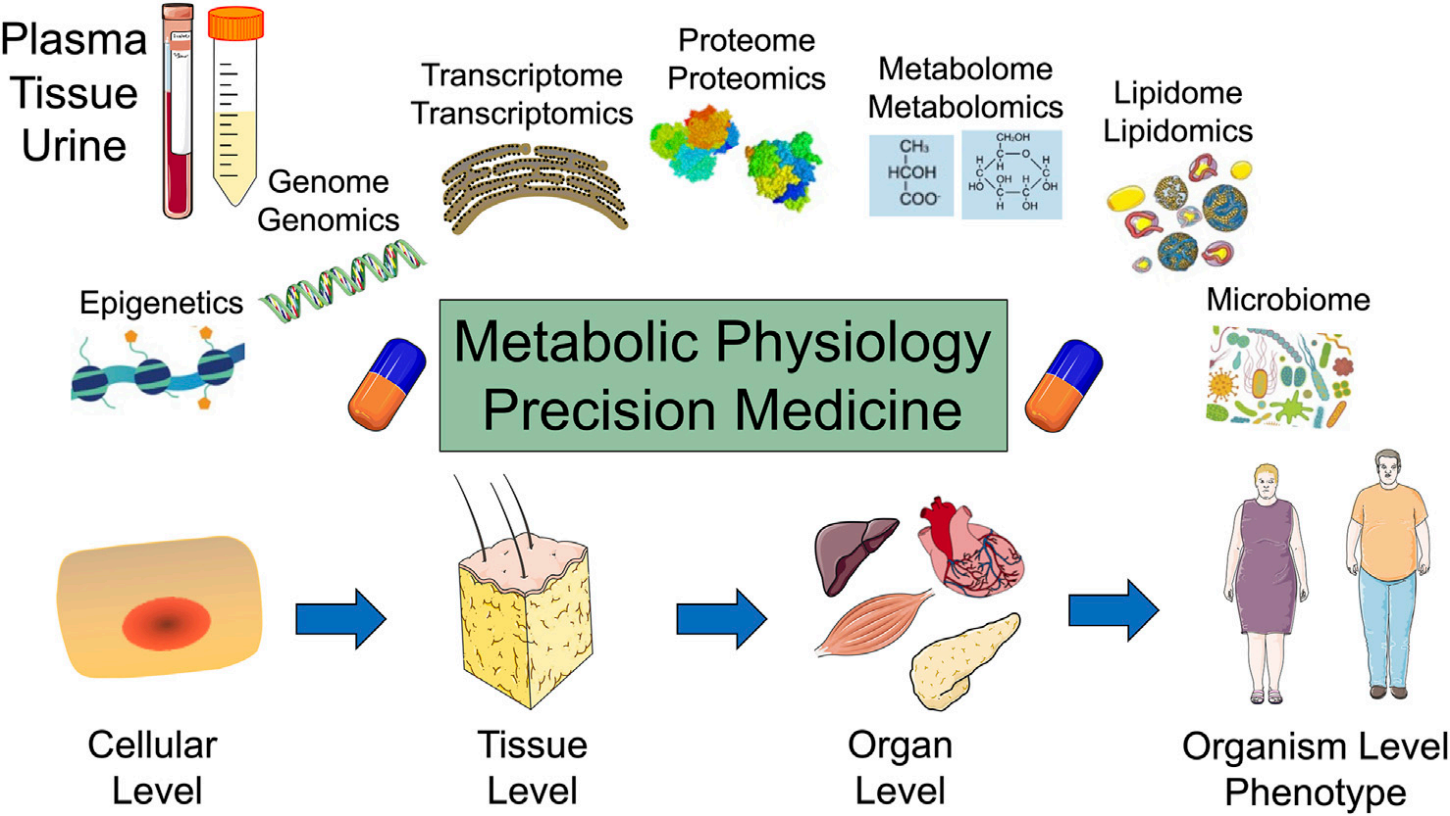
Metabolic physiology and the promise of precision medicine: Top: Plasma, tissue, and urine samples are analyzed for epigenetics, genomics, transcriptomics, proteomics, metabolomics, lipidomics, and microbiome. Bottom: Metabolism is evaluated at the cellular, tissue, organ, and organism levels.

Metabolites are key for cellular bioenergetics, biosynthesis, and redox demands. The regulation of gene expression by metabolites and metabolites as biomarkers for metabolic diseases are rapidly evolving metabolic physiology research areas (Lehr et al., 2012; Park et al., 2021). Cell metabolism can regulate gene expression through changing metabolic enzymes and their related metabolites. Epigenetic modifications are controlled by metabolites that interact with transcription factors or histone/DNA modifications. Disturbances in the regulation of gene expression or epigenetic modifications by cell metabolism can contribute to various diseases (Park et al., 2021). In addition, metabolomics is a powerful tool for the phenotyping and identification of molecular signatures and biomarkers. Emerging research is aimed at defining associations between metabolic biomarkers and chronic metabolic diseases, with a particular attention to cofounding factors such as age, sex, lifestyles, and co-morbidities (Hussain et al., 2021). A major challenge for metabolites and cell metabolism research is the ever-evolving complexity of data that includes intrinsic and extrinsic factors such as metabolomics, genetics, epigenetics, microbiota, longitudinal patient data, and environmental factors which have the promise to revolutionize precision and personalized medicine (Figure 1).

*Frontiers in Metabolic Physiology* is publishing articles that offer insight into metabolites, epigenetics, and metabolic diseases. Evaluation of placental metabolites in preeclampsia by metabolomics and lipidomics found perturbations in glycerophospholipid and glutathione metabolism which provided novel insight into the molecular mechanism underlying preeclampsia (Zhang et al., 2022). Another study determined global blood methylation patterns, the biochemical profile, and the metabolome of ewe lambs born from feed restricted dams. These findings demonstrated changes in methylation of genes in functional categories such as cellular processes, phosphorylation, nervous system, immunity response, or reproductive function are enriched significantly in lambs born to feed restricted dams (Andrés et al., 2021). Comparison of high-sugar and high-fat diets fed to rats and metabolic consequences were evaluated. A high-sugar, medium fat diet impacted insulin homeostasis and adipose secretory function whereas a high-fat diet primarily altered lipid and liver metabolism (Kotzé-Hörstmann et al., 2022). Neuronal mechanisms in fasted rats were the focus of another study. Findings in this study supported the hypothesis that in fed animals, increased adrenergic tone in dorsal raphe neurons inhibit feeding, while in fasted rats the adrenergic tone decreases and favors food intake (Flores et al., 2021). Another study focused on hepatic metabolomic profiling in a chronic intermittent hypoxia mouse model to identify altered metabolites and related metabolic pathways. Metabolites in amino acid metabolism, membrane transport, lipid metabolism, carbohydrate metabolism, nucleotide metabolism, and ferroptosis were identified as critical metabolites in chronic intermittent hypoxia induced liver injury (Chen et al., 2021). These are just a sampling of articles published in *Frontiers in Metabolic Physiology* that are tackling the challenge of understanding metabolism and metabolites in physiological and pathological states.

## Grand challenge: Organ regulation in metabolic physiology

Several organs play a central role in metabolic homeostasis by regulating synthesis, metabolism, storage, redistribution, and utilization of carbohydrates, lipids, and amino acids. Critical organs include the gastrointestinal tract, pancreas, liver, adipose tissue, and skeletal muscle. The gastrointestinal tract is responsible for the digestion and uptake of metabolites. Regulating digestive enzymes as well as glucose utilization through insulin secretions are key roles for the pancreas. Metabolic processes in the liver include lipogenesis, fatty acid oxidation, gluconeogenesis, cholesterol biosynthesis and elimination, and lipoprotein transport (Ponziani et al., 2015; Jones, 2016). Adipose tissue is highly dynamic and contributes to maintaining energy balance and storing excess energy. Skeletal muscle makes a substantial contribution to promoting energy efficiency by uptaking glucose to conduct contractile function essential for movement and life. Understanding mechanisms underlying metabolic adaptations at the organ level and organ to organ communication at the whole-body level will be the key foundation to design novel strategies to prevent and combat metabolic diseases.

There are several major challenges being tackled at the organ level that have been highlighted in *Frontiers in Metabolic Physiology*. One major challenge is hepatic glucose and lipid metabolism to maintain metabolic homeostasis. Rapidly rising disorders of hepatic glucose and lipid metabolism can contribute to diabetes, obesity and metabolic syndrome, fatty liver disease, and atherosclerotic cardiovascular diseases (O'Neill and O'Driscoll, 2015; Ponziani et al., 2015). Important research areas in liver metabolism include identifying novel regulators and pathways that lead to glucose or lipid metabolic dysfunction, interactions between the liver and other metabolic tissues, and discovery of therapeutic strategies to regulate hepatic glucose and lipid metabolism to manage metabolic diseases (Monroy-Ramirez et al., 2021). Another challenge is increasing the understanding of the mechanisms that underpin dysfunctional adipose tissue in metabolic diseases. Adipose tissue is composed of brown adipose that accrues and burns lipid and white adipose that stores excessive energy (Wang Z. et al., 2021). Adipose tissue cellular dysfunction can lead to altered energy expenditure and lipid accumulation and inflammation (Kawai et al., 2021). Addressing key issues relating to adipose tissue distribution and function, adipose tissue regulation at the genetic, epigenetic, and protein levels, and impact of procedures such as liposuction and bariatric surgery in relation to metabolic diseases are urgently needed. Skeletal muscle is another major site for energy metabolism that is disturbed in metabolic diseases. Critical key metabolism research areas include contractile function, insulin sensitivity, mitochondrial function, energy efficiency and exercise therapy. Lastly, a rapidly expanding area of metabolism research is the gut microbiome in maintaining metabolic homeostasis and immune balance (Daisley et al., 2021). Although the ability for the gut microbiome to maintain immune balance has been extensively investigated, major gaps exist in our knowledge of the mechanism by which the gut microbiome contributes to physiologic homeostasis and metabolism.

A better understanding of organ communication and metabolic changes and adaptations at the organism level is needed to combat metabolic diseases. The ability of the organism to adapt to changes in physical activity, diet variations, and environments with varying temperatures and humidity levels is critical to maintain metabolic homeostasis (Galgani and Fernández-Verdejo, 2021; Gorman et al., 2021). Organ to organ communication is necessary to properly adjust energy expenditure in these situations (Löffler et al., 2021; Wang F. et al., 2021). Major research challenges in metabolic adaptation include investigating brain adipose tissue communication, organ communication in calorie restriction, variation in temperature on organ communication, and changes to organ communication in response to exercise or high physical activity.

Regulation of metabolism at the organ level is also being addressed by scientific articles in *Frontiers in Metabolic Physiology*. A proteomic evaluation of endothelial progenitor cells involved in vascular repair revealed alterations in inflammatory protein metabolites in response to shear stress (Yu et al., 2022). Cardiovascular function has been the focus of additional studies. Impairment of ceramide-mediated instant endothelial cell membrane resealing was demonstrated in diabetes (Chen et al., 2022). A human study found that different metabolites and lipids have specific effects on diabetic cardiomyopathy (Hao et al., 2022). Novel techniques have also been developed to evaluate cardiovascular health in metabolic diseases in rodents (Reho et al., 2022). Adipose tissue of obese type 2 diabetic humans treated with pioglitazone was the focus of another study. Findings demonstrated that pioglitazone protects the immunometabolic health of adipocytes through selective remodeling of the glycerophospholipid pool, characterized by a decrease in lipids enriched for arachidonic acid, such as plasmanylethanolamines and phosphatidylinositols (Palavicini et al., 2021). At the other end of the species’ spectrum, studies examined molecular mechanisms mediating food-induced reinforcement in the model system *C. elegans*. Findings in this study demonstrated that glucose metabolism plays a key role in mediating both food-induced reinforcement and enhancement and implicate serotonergic signaling through the 5-HT4 receptor (Schwartz et al., 2021). These three studies highlight the wide range of studies being published around organ regulation in metabolic physiology.

## Grand challenge: The epidemic of metabolic diseases

**FIGURE 2:**
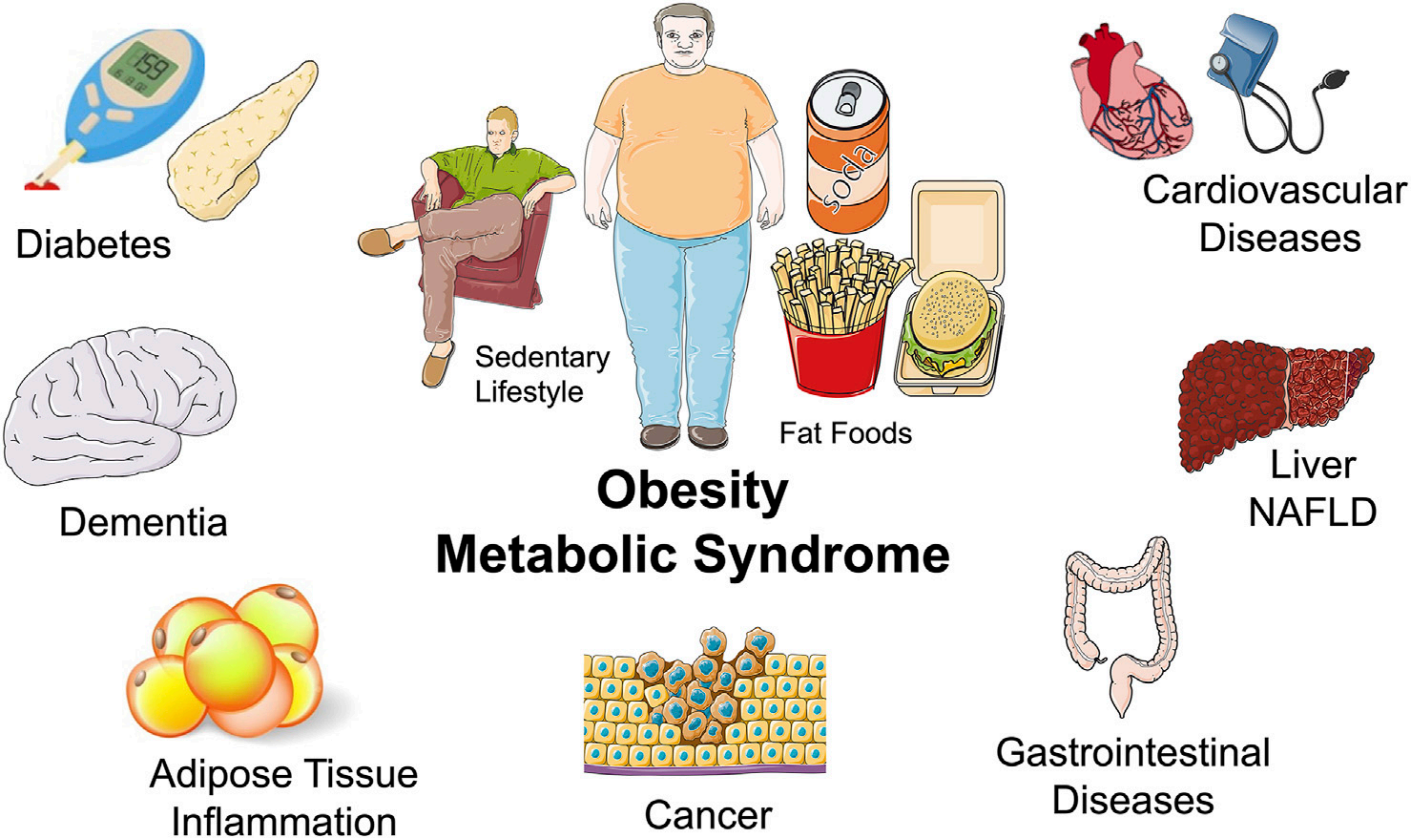
Obesity and metabolic syndrome and the epidemic of metabolic diseases: Middle: A sedentary lifestyle and a diet consisting of fat foods are major components to the obesity and metabolic disease epidemic. Surrounding: Diseases associated with obesity and metabolic diseases include diabetes, dementia, inflammatory diseases, cardiovascular diseases, liver disease such as non-alcoholic fatty liver disease (NAFLD), gastrointestinal diseases, and cancer.

There is an epidemic of metabolic diseases that place metabolic physiology research at the forefront. Technological advances in modern society have led to access to high calorie foods and decreased physical activity (O'Neill and O'Driscoll, 2015; Saklayen, 2018). This has led to a tripling in worldwide obesity since 1975 with 40% of adults being overweight (Iqbal et al., 2021). Obesity has been a primary driver for increased incidence of insulin resistance, inflammation, type 2 diabetes, non-alcoholic fatty liver disease (NAFLD), and cardiovascular diseases (O'Neill and O'Driscoll, 2015; Saklayen, 2018) (Figure 2). A more comprehensive understanding of mechanisms regulating metabolism at the cell, organ, and whole-body level is needed to discovery therapeutic approaches to prevent and treat metabolic diseases.

Major challenges in metabolic disease research start with the obesity epidemic. Obesity results from a combination of genetic factors, sedentary lifestyle, environmental influences, and diet (O'Neill and O'Driscoll, 2015; Saklayen, 2018). Increasing physical activity and decreasing caloric intake are first line interventions that have not achieved ideal outcomes (Saklayen, 2018; Gorman et al., 2021). As a result, a comprehensive understanding of mechanisms responsible for changes in metabolism is needed to prevent or treat obesity as well as the related diseases. Feeding behaviors can result in changes in metabolism and obesity. Several factors regulate feeding behaviors including macronutrients, taste, central nervous system and neural signals, and hormones like leptin and ghrelin (Nunez-Salces et al., 2021). These factors contribute to the control of food intake and energy balance that when dysfunctional alter feeding behaviors that result in obesity and metabolic diseases (Nunez-Salces et al., 2021). Key obesity research areas are understanding the pathogenesis of obesity, determining the impact of bariatric surgery, evaluating nutritional approaches to combat obesity, investigating neuro-behavioral aspects of obesity, and identifying signaling pathways that contribute to obesity.

Another major challenge associated with obesity is expansion of the adipose tissue that leads to adipokine and cytokine imbalances and a shift to a proinflammatory state (Kawai et al., 2021). The systemic low grade inflammatory state in obesity is characterized by abnormal activation of both the innate and adaptive immune systems (Kawai et al., 2021). Subsequently the global obesity epidemic has been paralleled by an increased incidence of autoimmune and immune-mediated diseases (Karczewski et al., 2021). Obesity driven inflammation also contributes to the pathophysiology of chronic diseases such as kidney, liver, neural, and cardiovascular diseases (O'Neill and O'Driscoll, 2015). Not known are the mechanisms by which obesity and adipose tissue contribute to the development of systemic inflammation and immune system activation or the mechanisms by which adipokines alter immune cell function in obesity. Overall, there are limited studies that have identified mechanistic links between obesity and related chronic diseases and autoimmune pathogenesis.

Diabetes is a metabolic disease that effects several organs that result in poor health and mortality (Shahcheraghi et al., 2021). Type 1 diabetes is characterized by a lack of insulin production and type 2 diabetes is associated with obesity and decreased physical activity which results in an ineffective use of insulin (Shahcheraghi et al., 2021). Although the discovery of insulin occurred 100 years ago and diabetes can be treated, diabetic complications such as neuropathy, retinopathy, nephropathy, strokes, and heart attacks remain common (Gupta et al., 2021; Shahcheraghi et al., 2021). In addition, the precise role of inflammation to diabetic complications remains unclear. Experimental studies suggest that an accelerated aging phenotype, coupled with a maladaptive immune response contribute to diabetic complications (Gupta et al., 2021; Shahcheraghi et al., 2021). Thus, there is an urgent need to understand the mechanisms and pathology by which diabetes and associated inflammation affects different organ systems. Research in this area will identify novel therapeutic targets for diabetic complications at the levels of transcription and translation, protein expression and activity, and cell and organ levels. Major challenges in diabetes include defining molecular mechanisms and pathways implicated in insulin metabolism, evaluating transcriptomics of high glucose on different cell types, defining the contribution of the innate immune response and NLRP3 inflammasome, understanding metabolic mechanisms that drive beta cell dysfunction, and defining metabolic processes in key insulin-target tissues.

NAFLD is a rapidly growing public health concern that occurs in 25% of the world population and is driven in large part by the obesity and type 2 diabetes epidemic (Caussy et al., 2021; Targher et al., 2021). Intriguingly, NAFLD can be as high as 75% in diabetic patients (Caussy et al., 2021; Targher et al., 2021). Non-alcoholic steatosis (NASH) is a type of NAFLD that is associated with inflammation and hepatocyte lipotoxicity which leads to liver fibrosis and cancer (Caussy et al., 2021; Targher et al., 2021). NASH is expected to become the leading cause for liver transplantation in the next decade (Nephew and Serper, 2021). Mechanisms that contribute to NAFLD and progression to NASH include regulation of *de novo* lipogenesis by acetyl-CoA carboxylase, regulation of bile acid signaling by farnesoid X receptor (FXR), or oxidative stress induced fibrogenesis and inflammation by apoptosis signal-regulating kinase 1 (ASK1) (Attia et al., 2021; Koo and Han, 2021). Although often associated with obesity and diabetes, understanding pathophysiological mechanisms at the cellular hepatocyte and organ liver levels that result in NAFLD and progression to NASH will be key to developing therapeutics.

Major challenges to the epidemic of metabolic diseases are the focus of several publications in *Frontiers in Metabolic Physiology*. Studies in mice with type 2 diabetes have revealed metabolites involved in diabetic kidney disease. Metabolomic analysis of kidneys revealed five dysregulated rate-limiting enzymes related to altered metabolic pathways involved in the progression of diabetic kidney disease (Linnan et al., 2021). Likewise, mechanisms for accelerating metabolic diseases have been explored. Findings in mice fed a high fat diet found that toll-like receptor 7 (TLR7) stimulation enhanced dysglycemia and hyperinsulinemia in mice (Kakalij et al., 2022). Human genetic studies revealed a variant in a metal ion transporter ZIP8 (SLC39A8) that leads to enhanced insulin resistance (Verouti et al., 2022). Kidney disease in obese rats treated with lisinopril was the focus of another study. Lisinopril significantly decreased glomerular injury and renal inflammation in obese rats (Brown et al., 2021). Human studies have also been revealed changes in metabolism that are linked to metabolic diseases. Findings from a study on metabolic patients demonstrated that serum fatty acid binding protein 4 (FABP4) is associated to liver steatosis (Rodríguez-Calvo et al., 2021). Likewise, type 2 diabetes patients have been investigated for associations with NAFLD. Findings revealed a monocyte to high-density lipoprotein cholesterol ratio as an inflammatory biomarker to assess NAFLD in type 2 diabetic patients (Jia et al., 2021). The human gut microbiome and effects on metabolites in liver and metabolic diseases are providing novel diagnostics and potential approaches to treat metabolic diseases (Lai et al., 2022; Tayachew et al., 2022). Future experimental studies that evaluate animal models as well as humans will be essential for addressing the epidemic of metabolic diseases.

## Grand challenge: Metabolic adaptation to the environment

Hibernation is a physiological state that allows an animal to adapt to extreme temperature challenges and inadequate food and water. Mammals that hibernate slow their metabolism, reduce their body temperature resulting in a state of hypothermic torpor. This metabolic rate reduction occurs on a whole-body scale due to molecular interactions that change the physiology of cells, tissues, and organs (Mohr et al., 2020; Giroud et al., 2021). Hibernation involves bouts of torpor with periodic intermittent bouts of euthermia arousal. There are both fat- and food-storing species that have different metabolic and digestive adaptations that occur during hibernation (Andrews, 2019). Our understanding of how hibernators change metabolism at the molecular, cellular, organ, and organism levels is leading to innovations in human medicine (Bertile et al., 2021). As an example, hibernating bears do not develop azotemia despite anuria and reductions in renal blood flow and glomerular filtration rate (Stenvinkel et al., 2018). Evaluation of metabolic changes in that occur in bears from summer to winter could provide novel therapeutics for chronic kidney disease and kidney preservation for transplantation (Ratigan and McKay, 2016; Stenvinkel et al., 2018).

Mammals generate their own heat to maintain constant body temperature despite variations in environmental temperature. This process of thermogenesis by mammals exposed to cold has been extensively studied (Reinisch et al., 2020). Understanding the mechanisms of adaptation to cold could assist in the treatment for obesity-related diseases (Chen et al., 2020). There has been a focus on brown adipose tissue that could play an important contribution to non-shivering thermogenesis in humans (Chen et al., 2020; Levy and Leonard, 2022). Cold exposure activates brown adipocytes that dissipate glucose and fatty acids. Intriguingly, cold exposure studies have suggested a role for fibroblast growth factor-21 (FGF21) that increases in cold acclimated mice to maintain body temperature (Klein Hazebroek and Keipert, 2020). The fact that FGF21 is secreted by brown fat or liver in the cold has led to speculation as a target for obesity-related diseases (Klein Hazebroek and Keipert, 2020). Another focus has been the impact of n-6/n-3 polyunsaturated fatty acid ratio on energy expenditure and adaptive thermogenesis as a strategy for preventing obesity (Fan et al., 2019). Although humans can control their environmental temperature, understanding mechanisms by which thermogenesis and adipocytes change in response to cold exposure could lead to novel therapies for metabolic syndrome and other diseases.

Metabolic adaptation to the environment by animals and humans can result in new treatments for metabolic related disorders, as well, as other diseases. *Frontiers* has been publishing articles on hibernation and cold exposure (Klein Hazebroek and Keipert, 2020; Bertile et al., 2021). *Frontiers in Metabolic Physiology* encourages submission of research topics, research articles, and review articles on metabolic changes during hibernation and in response to extreme temperature environments.

## Future directions

The frightening increase in metabolic diseases worldwide underscores the need to understand mechanisms that regulate metabolism at the cell, organ, and whole-body levels. *Frontiers in Metabolic Physiology* will continue to address issues related to our basic understanding of metabolism and metabolites, metabolic regulation at the organ level, and the metabolic disease epidemic. There needs to be greater emphasis on the fundamental differences in metabolic homeostasis between males and females. Sex differences in metabolic homeostasis, diabetes, and obesity could lead to sex-based therapeutic approaches for metabolic diseases. A rapidly evolving area in metabolism is evaluation of genetics and bioactive metabolites using single cell and tissue omics approaches. *Frontiers in Metabolic Physiology* has already published findings using omic approaches (Hao et al., 2022; Li et al., 2022; McRae et al., 2022). The ability to handle and analyze this data in a manner that allows for clinical translation will be critical. Current and future Research Topics will focus on rising star investigators, novel methods and techniques, omics, sex differences, environment and climate change on metabolism, metabolism in cancer and the metabolic disease epidemic. *Frontiers in Metabolic Physiology* provides a forum to share knowledge and progress in the field and will be at the research forefront by publishing scientific articles and reviews that address grand challenges.
